# Utilising Teledentistry for Interdisciplinary Oral Assessment in Older Patients: An International Cross-Sectional Survey

**DOI:** 10.3390/dj14060367

**Published:** 2026-06-15

**Authors:** Panagiota Chatzidou, Olga Naka, John Fanourgiakis, Eftychia Tsanana, Christos Armeniakos, Lisa Christina Pezarou, Aggelos Sfyrakis, Vassiliki Anastasiadou

**Affiliations:** 1 Department of Prosthodontics, School of Dentistry, Aristotle University of Thessaloniki, 54124 Thessaloniki, Greece; naka@dent.auth.gr (O.N.); chrisarmenuni@gmail.com (C.A.); lpezarou@gmail.com (L.C.P.); sfyrakispersonal@gmail.com (A.S.); vanasta@dent.auth.gr (V.A.); 2 Department of Management Science and Technology, Hellenic Mediterranean University, 72100 Agios Nikolaos, Greece; jfanourgiakis@hmu.gr; 3 Department of Economics, University of Macedonia, 54124 Thessaloniki, Greece; ef.tsanana@gmail.com

**Keywords:** teledentistry, intraoral scanning, older adults, interdisciplinary care, prosthodontics, geriatric dentistry

## Abstract

**Background/Objective**: The increasing global population of older adults presents significant challenges for oral healthcare, particularly regarding the management of chronic conditions and prosthetic rehabilitation. Teledentistry, combined with intraoral scanning, offers a promising solution to enhance access, interdisciplinary collaboration, and clinical outcomes in geriatric populations. This study aimed to evaluate the utilisation of intraoral digital scanning within teledentistry for interdisciplinary oral assessment of older patients. Specifically, it investigated current clinical practices, collaboration among healthcare professionals, and perceptions regarding the effectiveness, challenges, and future potential of teledentistry in prosthodontic care. **Methods**: An analytical cross-sectional survey was conducted among 84 healthcare professionals, including dentists, prosthodontists, and postgraduate students, recruited via an international network. Participants completed a 40-item electronic questionnaire covering demographics, clinical practice, digital technology use, interdisciplinary collaboration, and attitudes toward research and innovation. Descriptive statistics summarised responses, and inferential analyses, including chi-square tests and Spearman correlations, examined associations between career stage, technology adoption, and interdisciplinary practices. **Results**: Early-career professionals demonstrated the highest adoption of intraoral scanning (76.3%), while mid-career adoption was lowest (28.6%). Sustained usage significantly increased after one year of adoption (93.8%). While 91.7% of respondents valued interdisciplinary care, active collaboration remained limited. Cost, technical barriers, and training gaps were identified as primary obstacles. Professionals perceived intraoral scanning as beneficial for prosthodontic outcomes and chronic inflammation management, though adoption was influenced by experience, systemic factors, and financial support. **Conclusions**: Teledentistry integrated with intraoral scanning has substantial potential to improve geriatric oral healthcare. Successful implementation depends on structured training, financial investment, and promotion of interdisciplinary collaboration. Future longitudinal and multicenter studies are warranted to evaluate clinical, economic, and patient-centred outcomes, supporting sustainable digital transformation in geriatric dental care.

## 1. Introduction

The growing population of older adults worldwide poses significant challenges for healthcare systems, particularly in relation to oral health. Older adults are at increased risk of oral diseases, often compounded by chronic conditions such as hypertension, diabetes, and Alzheimer’s disease, which adversely affect oral health and the ability to adapt to prosthetic restorations [1]. In this context, teledentistry has emerged as a promising approach to improving the quality of dental care for older adults [2].

Despite numerous public and private initiatives, disparities in oral care access persist, particularly among older adults. Teledentistry has gained recognition as a cost-effective solution to reduce these inequalities, improve patient outcomes, enhance provider efficiency, and support sustainable dental care delivery. It is broadly defined as using technology to provide remote oral healthcare between patients and providers or among healthcare professionals, with objectives that include improving access, reducing disparities, alleviating economic burdens, and fostering interdisciplinary collaboration [3].

Teledentistry has become a significant advancement in oral healthcare delivery, improving access to dental services for older adults, particularly those with mobility limitations or living in geographically underserved areas [4]. Through synchronous and asynchronous modalities, including digital imaging and recorded video-based assessment, it enables remote oral health evaluation while reducing the need for in-person clinical attendance [5]. Its clinical utility has been demonstrated in the early detection of oral malignancies [6], monitoring oral complications associated with oncologic treatment [7], and supporting oral function and nutritional outcomes through tele-education and mobile health interventions [8,9]. The integration of intraoral digital scanning further enhances these capabilities by enabling precise digital capture of oral structures and facilitating specialist consultation in prosthodontic care [10].

Teledentistry also offers organisational and economic advantages. Evidence from institutional settings, including nursing homes, suggests that it may be a cost-effective alternative to conventional care, improving accessibility and convenience [11]. Its effectiveness has also been reported in the management of dental emergencies through remote triage and referral [12,13]. In addition, teledentistry supports interdisciplinary collaboration among dental professionals and other healthcare providers, contributing to more integrated and patient-centered care for older adults [14,15]. Remote educational interventions may further enhance oral hygiene practices, autonomy, and adherence to oral healthcare recommendations [8,16].

Despite these benefits, several barriers remain, including limitations in digital literacy, technological accessibility, and infrastructure, particularly in resource-constrained settings [11,17]. Moreover, current evidence is constrained by methodological heterogeneity and limited high-quality economic and clinical evaluations, highlighting the need for more rigorous research to support evidence-based implementation [2,18].

Although teledentistry shows considerable promise, evidence regarding its integration with intraoral digital scanning in geriatric prosthodontic care remains limited. Data on its feasibility, clinical applicability, and perceived value in prosthetic rehabilitation for older adults are scarce.

This study aimed to assess the use of intraoral digital scanning within teledentistry for the interdisciplinary oral evaluation of older adults, as well as to investigate healthcare professionals’ knowledge, attitudes, and clinical practices regarding its application. Furthermore, the study examined the extent and effectiveness of interdisciplinary collaboration, explored professionals’ perceptions of the effectiveness, challenges, and future potential of teledentistry, and analysed the association of these factors with their demographic and professional characteristics (qualifications and clinical experience).

## 2. Materials and Methods

### 2.1. Study Design and Participants

This analytical cross-sectional study examined healthcare professionals’ knowledge, attitudes, confidence, clinical practices, perceived barriers, and training needs regarding teledentistry and interdisciplinary approaches to the care of older adults.

The study population comprised dentists, postgraduate dental students, and other healthcare professionals with clinical involvement in older adult care. Participants were recruited through an international professional and alumni network. Purposive sampling captured a broad range of professional roles, practice settings, and geographic regions. Eligibility criteria included active clinical involvement in older adult care, English-language proficiency, internet access, and provision of informed consent (Appendix A). Although participants were drawn from multiple countries and healthcare settings, the predominance of European respondents may limit the wider generalisability of the findings.

### 2.2. Questionnaire Development and Data Collection

The questionnaire was developed following a structured review of the literature on teledentistry, gerodontology, and prosthodontic care. Content validity was established through expert review by specialists in prosthodontics, gerodontology, and digital dentistry.

The final instrument comprised 40 items covering demographic and professional characteristics, clinical practice patterns, utilisation of digital technologies, interdisciplinary collaboration, perceptions of teledentistry, and future perspectives on research and innovation. A combination of Likert-scale, dichotomous, multiple-choice, and limited open-ended questions was used to capture both quantitative and qualitative data (Table 1). The estimated completion time was 20–25 min.

Data was collected anonymously using a structured, self-administered online questionnaire (Appendix B) through a secure online platform. Prior to implementation, the questionnaire was pilot tested among postgraduate students to assess clarity, comprehensibility, and feasibility.

### 2.3. Study Variables and Outcome Measures

Independent variables included age, sex, educational background, speciality, years of clinical experience, practice setting, geographic region, patient age profile, and level of engagement in geriatric care.

Outcome measures were derived from questionnaire responses (Table 1). They included reported clinical practices, use of intraoral scanning technologies, approaches to oral health assessment, interdisciplinary collaboration, perceptions of teledentistry, and attitudes towards research and innovation. Additional outcomes included measures of knowledge, confidence, perceived barriers, and training needs relating to the implementation of teledentistry and collaborative care models.

### 2.4. Statistical Analysis

Statistical analyses were conducted using IBM SPSS Statistics (Version 27.0.1.0). Descriptive statistics were used to summarise participant characteristics and questionnaire responses. Categorical variables were presented as frequencies and percentages, whereas ordinal variables were summarised using appropriate measures of central tendency and dispersion.

Given the predominantly ordinal nature of the data and the absence of assumptions required for parametric testing, non-parametric statistical methods were employed. Associations between categorical variables were examined using Pearson’s chi-square test, with categories combined where necessary to satisfy test assumptions. Fisher’s Exact Test was applied to 2 × 2 contingency tables with low expected cell frequencies. Relationships between ordinal variables were assessed using Spearman’s rank-order correlation. Comparative analyses were performed across professional groups and selected demographic and professional characteristics, including level of experience, geographic region, and practice setting. All statistical tests were two-tailed, and statistical significance was established at *p* < 0.05.

### 2.5. Reliability Assessment

Internal consistency was assessed only for conceptually related item clusters, as the questionnaire comprised multidimensional constructs and heterogeneous variable types, rendering the calculation of a single overall reliability coefficient inappropriate. Negatively worded items were reverse-coded prior to analysis.

Reliability was evaluated using Cronbach’s alpha for Likert-scale measures and the Kuder–Richardson 20 (KR-20) coefficient for dichotomous variables. Two composite indices demonstrated acceptable internal consistency and were retained for further analysis: the Willingness to Participate in Research Index (Questions 24 and 38; KR-20 = 0.823) and the Interdisciplinary Approach and Prosthodontics Index (Questions 21 and 22; Cronbach’s α = 0.723). All remaining questionnaire items were analysed individually.

### 2.6. Ethical Considerations

Ethical approval was obtained from the Research Ethics Committee of the School of Dentistry, Aristotle University of Thessaloniki (protocol code 279 the 20 March 2025). The study was conducted in accordance with the Declaration of Helsinki. Participation was voluntary, and informed electronic consent was obtained from all respondents prior to data collection. All data were anonymised, securely stored, and processed in compliance with the General Data Protection Regulation (GDPR).

## 3. Results

### 3.1. Descriptive Analysis

#### 3.1.1. Demographics

The study sample consisted of 84 healthcare professionals, with the majority (90.5%) based in Europe. Age distribution showed that 50.0% were aged 20–39, while 33.3% were between 50–59 years. Gender was nearly balanced (52.4% male, 47.6% female). Most participants held a DDS/DMD or a postgraduate degree.

Regarding speciality, 36.9% were general dentists and 26.2% prosthodontists. Smaller proportions (6–7%) included endodontists, oral surgeons, and nurses, while 1–2% represented highly specialised fields (e.g., implantology, periodontology, gerodontology).

Professional experience was polarised: 33.3% had 0–5 years, while 38.1% had over 20 years. In terms of employment, 42.9% worked in private practice and 25.0% combined private and academic roles. A large majority (90.4%) practised in Europe. Additionally, 71.4% reported that patients aged ≥65 comprised up to 50.0% of their patient base. Demographic characteristics of the study sample are summarised in Table 2.

#### 3.1.2. Clinical Practice

Monitoring of geriatric patients was frequent, with 58.3% reporting daily and 32.1% weekly follow-up. The most common oral health concerns were tooth loss and periodontal disease, often co-occurring, with some patients also presenting xerostomia.

Chronic inflammation was perceived negatively by 89.3% of respondents (42.9% significantly adverse, 46.4% moderately adverse). Assessment methods included clinical examination with radiographs (52.4%), clinical examination alone (15.5%), and a combination including intraoral scanning (14.3%).

Importantly, 94.0% agreed that chronic inflammation negatively affects prosthetic treatment outcomes. Clinical practice characteristics related to geriatric patients are presented in Table 3.

#### 3.1.3. Utilisation of Intraoral Scanning Technology

Most participants (61.9%) had used intraoral scanning for less than one year, while 27.4% had 1–3 years of experience. Regarding its impact, 45.2% reported no effect on inflammation management, while 31.0% noted significant improvement and 23.8% moderate improvement. Overall, 54.8% perceived a positive effect. In CAD/CAM prosthetics, 33% most commonly fabricated fixed partial dentures and implant-supported prostheses.

A full range of restorations was reported by 13.1%, while 11.9% focused solely on implant-supported prostheses. Notably, 17.9% did not use CAD/CAM for any restorations. Advantages included reduced chair time (27.4%), while 15.5% valued all benefits equally. Conversely, 11.9% saw no advantages. Key barriers were cost (23.8%), technical limitations (17.9%), and both combined (13.1%). Results regarding the utilisation of intraoral scanning and CAD/CAM technology are shown in Table 4.

#### 3.1.4. Interdisciplinary Approach

Most respondents did not collaborate with other specialists (Table 5). However, 91.7% considered interdisciplinary care important or very important. Additionally, 90.4% emphasized the role of prosthodontics in geriatric health (33.3% critical, 57.1% important).

#### 3.1.5. Future Research and Innovations

A majority (83.3%) believed intraoral scanning would have a moderate to high impact in gerodontology. However, only 63.1% expressed willingness to participate in related research.

Key research priorities included long-term prosthetic outcomes and interdisciplinary care are presented in Table 6. Most respondents perceived intraoral scanning as having either a high (44.0%) or moderate (39.3%) impact on gerodontology. Nearly two-thirds (63.1%) expressed interest in participating in future research. Regarding research priorities, interdisciplinary approaches emerged as the most frequently cited theme (60.8%), followed by long-term treatment outcomes (33.3%), patient collaboration (28.6%), and digital technologies such as intraoral scanning and CAD/CAM systems (25.0%). Other priorities accounted for 30.9% of responses. Because respondents could select multiple themes, percentages exceed 100%.

#### 3.1.6. Professional Perspectives and Confidence

Confidence levels were moderate for 54.8% and high for 25.0% in diagnosing inflammatory conditions. Despite this, 95.2% reported challenges in geriatric care.

Interest in education was evident: 23.8% favored continuing education, 22.6% preferred combined methods (courses, workshops, online resources, collaboration), and 16.7% emphasised education plus collaboration. Results regarding Professional perspectives and confidence are summarised in Table 7.

#### 3.1.7. Patient Beliefs

According to professionals, 41.7% of elderly patients recognise oral health’s importance, while 30.9% consider it secondary, and 19.0% remain neutral.

Treatment decisions were influenced primarily by cost and functionality. Specifically, 14.3% cited cost alone, 22.6% cost plus functionality, and 20.2% included aesthetics. These findings highlight the multifactorial nature of decision-making (Table 8).

#### 3.1.8. Last Questions

Strategic priorities and system needs are included in Table 9. Most respondents (76.2%) identified awareness, accessibility, and specialised geriatric services as key strategic priorities for improving geriatric oral healthcare, while 23.8% favored comprehensive or alternative approaches.

Regarding effective care delivery, 57.1% emphasised specific factors such as financial support, training, and collaboration, whereas 42.9% supported a broader, comprehensive approach or identified other factors.

These findings underscore the importance of both targeted interventions and integrated system-level strategies to enhance oral healthcare for older adults.

#### 3.1.9. Evaluation

More than half of respondents were satisfied with their capacity to provide geriatric care (58.3%) and considered intraoral scanning effective in clinical practice (51.2%). Nearly two-thirds (63.1%) favoured a multidimensional approach to assessing prosthetic treatment success, while 51.2% preferred formal educational activities for continuing professional development.

Financial and access-related challenges were identified as the predominant barriers to geriatric oral healthcare (71.4%). Most participants reported observing changes in patient needs over time (82.1%) and expressed willingness to participate in future research (77.4%). Responses regarding the evaluation of clinical Practice are presented in Table 10.

### 3.2. Inferential Statistics

To address the research questions and the primary scope of this study, various statistical measures were employed to identify significant associations and correlations between certain variables. Due to sample size limitations and the necessity of collapsing categories to meet test assumptions, these inferential findings should be interpreted cautiously and viewed as exploratory.

#### 3.2.1. Career Stage and Technology Adoption

Pearson’s chi-square test of independence was performed to examine the relationship between career stage (question 5) and the current use of intraoral scanning technology (question 14).

To ensure statistical robustness, satisfy the assumptions of the test and address low expected cell counts, the original categories for years of experience were collapsed into three groups: early career (0–10 years), mid-career (11–20 years), and experienced (more than 20 years). The association between career stage and technology use was found to be statistically significant (chi-square = 10.03, *p* = 0.007). According to the relevant contingency table, early-career professionals demonstrated the highest adoption rate (76.3%), followed by those with more than 20 years of experience (59.4%). In contrast, the mid-career group reported the lowest adoption rate at 28.6%. While these results suggest a potential non-linear relationship, where adoption is highest at the beginning of the career, dips significantly during the mid-career phase, and rises again among the most experienced staff, this finding should be interpreted cautiously due to the loss of data granularity from collapsing the categories.

#### 3.2.2. Technology Adoption and Duration of Use

To further evaluate intraoral scanning technology adoption (question 14), the duration of technology use was collapsed into a binary variable—initial adoption (less than 1 year) and sustained adoption (1 year or more). This categorisation was necessary to satisfy the minimum expected cell count assumption (lowest expected count = 12.19). A Pearson’s chi-square test revealed a highly significant association between the duration of use and current adoption status (chi-square = 20.10, *p* ≤ 0.001). A review of the frequencies suggested a noticeable shift in retention once the one-year threshold is crossed. Among participants who used this technology for less than a year, the majority (57.7%) are no longer current users. Conversely, 93.8% of those who utilised the technology for a year or more remain current users.

This indicates a potential retention threshold, suggesting that user attrition may occur almost entirely within the first twelve months of adoption. However, further studies with larger samples are needed to confirm this dynamic.

#### 3.2.3. Perception of Inflammaging and Prosthodontic Treatment Beliefs

To examine the relationship between participants’ perceptions regarding the impact of chronic inflammation (question 11) on oral health and their beliefs concerning its effect on the success of prosthodontic treatments (question 13), categories were collapsed into 2 categories: Adverse vs. Neutral/Positive impact and Yes vs. Unsure. Due to small, expected cell counts, Fisher’s Exact Test was employed. The analysis indicated no statistically significant association between the perceived severity of inflammation and beliefs regarding prosthodontic treatment outcomes (*p* = 0.441). Furthermore, the correlation remained very weak and non-significant (Spearman’s r = 0.076, *p* = 0.495). This result should be interpreted cautiously, as the high homogeneity within the sample limits the statistical power of the analysis: 89.3% of participants categorised the impact of inflammation as adverse, and 94.0% affirmed the belief that it affects prosthodontic success. This lack of variance and the small cell sizes likely constrained the statistical power of the test to identify a distinct correlation.

#### 3.2.4. Interdisciplinary Approach and Perceived Importance

A Pearson chi-square test was conducted to investigate the relationship between active professional collaboration (question 20) and the perceived importance of such synergy (question 21). The analysis revealed a statistically significant association between the two variables (chi-square = 27.524, *p* < 0.001). Participants who actively collaborate with other professionals were significantly more likely to rate the importance of collaboration as “Very Important” compared to those who did not. Supporting this finding, a strong positive correlation was observed (Spearman’s r = 0.548, *p* < 0.001), suggesting that active engagement in interprofessional collaboration is closely linked to a higher valuation of its significance.

#### 3.2.5. Strategies for Ageing Populations and Systemic Factors

Finally, an exploratory analysis was performed to explore the relationship between the strategies for effectively catering to the ageing population (question 31) and the factors considered necessary for older patients (question 32). Although an initial chi-square reached statistical significance (chi-square = 176.31, *p* < 0.001), this result must be interpreted with extreme caution, as 98.5% of the cells had expected counts of less than 5. This violation of the test’s foundational assumptions is attributed to the high fragmentation of response categories, as participants identified a wide variety of multifaceted solutions.

Given this limitation, the relationship is better interpreted through descriptive trends rather than rigid inferential testing. Descriptively, “Financial support” emerged as the most frequently cited requirement for addressing needs. Regarding primary goals, “Increasing awareness and education” and “Improving accessibility to dental care” were the most identified strategies among participants.

To further investigate without relying on chi-square assumptions, Spearman’s rank-order correlation analysis was conducted. This analysis confirmed that there is no significant relationship between the fragmented goals and factors (r = 0.065, *p* = 0.551), suggesting that participants view the necessary systemic factors as universally applicable baseline needs across all identified strategic goals.

## 4. Discussion

Mapping the Digital Transition in Gerodontology: Career Stage Determinants, Retention Thresholds, and Systemic Barriers.

### 4.1. The U-Shaped Curve of Digital Adoption: From Early-Career Native to Seasoned Expert

The findings of the present study reveal a distinct nonlinear, U-shaped relationship between career stage and digital technology adoption among healthcare professionals. While the high adoption rate among early-career practitioners (76.3%) aligns with the well-established “digital native” hypothesis—suggesting a natural inclination toward integrating digital workflows—the substantial adoption observed among experienced clinicians (59.4%) challenges conventional assumptions of late-career technological resistance. This bimodal distribution suggests that while younger professionals adopt technology as a foundational element of their training, seasoned experts strategically leverage digital tools to optimize efficiency and manage established clinical workloads. Conversely, the significantly lower adoption rate among mid-career professionals (28.6%) points to potential time constraints or practice-building pressures that may restrict the window available for clinical workflow restructuring.

These findings are consistent with the growing global uptake of digital technologies and teledentistry among oral healthcare professionals [19]. Increasing awareness and knowledge regarding teledentistry have been documented among both dentists and patients [20], while systematic reviews have highlighted its potential role in improving access to oral healthcare services for older adults [21]. Implementation initiatives specifically targeting geriatric populations further demonstrate the feasibility of integrating patient-centered digital oral healthcare models into routine practice [22]. In addition, studies comparing in-person and remote examinations have reported encouraging levels of diagnostic agreement and clinical decision-making accuracy [23], while investigations of teledentistry-based caries detection have demonstrated reliable diagnostic performance [24,25]. Broader reviews of innovations in geriatric oral healthcare have similarly emphasized the expanding role of digital technologies in supporting care delivery for aging populations [26].

The increasing relevance of digital technologies extends beyond clinical convenience. Sustainable healthcare frameworks have identified teledentistry as a mechanism capable of improving access while reducing barriers associated with geographic distance and resource limitations [27]. Community-based experiences involving older adults in underserved populations have further demonstrated the practical value of remote oral healthcare delivery [28]. Systematic reviews and meta-analyses have reported positive outcomes for oral health promotion, prevention, and patient monitoring through teledentistry and mobile health approaches [29]. Likewise, patient-centered screening and assessment models increasingly utilize digital technologies to facilitate early intervention and continuity of care [30].

Educational preparedness may also contribute to the observed adoption pattern. Recent evidence suggests that exposure to digital technologies during training improves readiness for implementation in clinical practice [31]. Structured teledentistry education programs have been shown to increase practitioner confidence and facilitate integration into routine workflows [32]. Similar trends have been observed among dental professionals internationally, with increasing awareness and acceptance of teledentistry [33]. Global reviews further highlight the growing range of clinical applications supported by digital oral healthcare technologies [34], while recent conceptual work has helped clarify the evolving scope and definition of teledentistry within contemporary dental practice [35].

Crucially, the inferential analysis identifies a definitive “retention threshold” at the 12-month mark. The dramatic divergence in sustained usage—where 57.7% of users who utilized the technology for less than a year discontinued its use, compared to an overwhelming 93.8% retention rate among those surpassing the one-year mark—indicates that implementation barriers are heavily front-loaded. Once the initial learning curve and immediate workflow disruptions are overcome, digital technology transitions from an experimental modality into an indispensable asset in professional practice. This interpretation is supported by studies demonstrating that successful long-term adoption depends not only on access to technology but also on training, usability, perceived usefulness, and organisational support mechanisms [31,32,33,34,35].

### 4.2. Clinical Reality vs. Diagnostic Inertia: The Inflammaging Ceiling Effect and Digital Lag in Gerodontology

Regarding clinical practice in gerodontology, a near-universal consensus emerged regarding the pathological significance of chronic inflammation, with 89.3% of respondents recognising its adverse effects and 94.0% affirming its negative impact on prosthetic outcomes. Interestingly, inferential testing yielded no statistically significant correlation between the perceived severity of inflammation and specific prosthodontic treatment beliefs (Spearman’s r = 0.076, *p* = 0.441). Rather than indicating a true lack of clinical association, this result is primarily attributable to a profound “ceiling effect” driven by the high homogeneity of the sample. Because most practitioners already hold an advanced understanding of the biological mechanisms linking chronic inflammation to prosthetic failure, statistical variance was constrained.

However, a notable discrepancy was observed between this advanced theoretical understanding and actual diagnostic behaviour. Despite acknowledging the clinical burden of inflammation, over two-thirds of the sample continue to rely exclusively on traditional diagnostic methods, such as conventional clinical examinations alone (15.5%) or clinical examinations combined with radiography (52.4%). Only 14.3% reported integrating advanced digital tools, such as intraoral scanning, into their diagnostic paradigm, highlighting a critical lag between recognition of complex geriatric pathologies and adoption of modern multidimensional monitoring technologies.

This implementation gap is particularly noteworthy given the increasing emphasis on interdisciplinary and technology-assisted approaches to oral healthcare among older adults [36]. Recent reviews have demonstrated substantial agreement between virtual and conventional examinations in geriatric populations [37], while pilot investigations in aged-care facilities have reported encouraging levels of diagnostic accuracy using teledentistry approaches [38]. Additional studies have confirmed the effectiveness of asynchronous teledentistry for detecting dental caries [39] and have demonstrated the feasibility of smartphone-based diagnostics among geriatric patients [40]. Multicenter investigations have further supported the accuracy of mobile health assessments using photographic documentation [41]. Similar findings have been reported for oral cancer screening in low-resource settings [42] and for video-based teleconsultations used in the diagnosis of oral lesions [43]. Collectively, these findings suggest that the observed lag reflects implementation barriers rather than limitations in available technology.

### 4.3. The Self-Reinforcing Collaborative Gap

A similarly pronounced implementation gap was identified concerning interdisciplinary care. Although 91.7% of participants characterised interprofessional synergy as important or very important for geriatric oral health, 60.7% reported that they do not currently engage in active collaboration with other specialists. This discrepancy suggests that barriers to collaborative practice are rooted in systemic and structural deficiencies rather than individual professional reluctance.

Evidence from integrated oral healthcare models in aged-care facilities has demonstrated that collaboration between oral health practitioners and telehealth-supported services can improve access to care and continuity of treatment for older adults [44]. Likewise, studies examining relationships between dental professionals and non-dental healthcare providers have highlighted the importance of structured interprofessional networks for managing complex patient needs [45]. Similar observations have been reported in palliative care settings, where practitioners recognise the necessity of integrating oral health into broader patient management frameworks [46].

Crucially, the inferential analysis revealed a strong positive correlation between active collaboration and the valuation of its significance (r = 0.548, *p* < 0.001). This finding underscores a “learning-by-doing” or self-reinforcing phenomenon: the clinical utility and multifaceted benefits of interdisciplinary care—such as managing polypharmacy or complex systemic comorbidities in geriatric patients—only become fully realised once the practitioner is actively engaged in the process. Consequently, fostering collaborative frameworks early and consistently within professional networks is essential to transition clinicians from passive ideological agreement to active, integrated clinical practice. The literature increasingly supports this perspective, demonstrating that exposure to collaborative care environments enhances appreciation of interdisciplinary contributions and improves patient-centered outcomes [44,45,46].

### 4.4. The Confidence Paradox in Geriatric Care: Individual Competence vs. Systemic Complexities

The data also highlight a compelling “confidence paradox” within professional perspectives. While most practitioners reported moderate (54.8%) to high (25.0%) confidence in diagnosing inflammatory conditions, an overwhelming 95.2% acknowledged facing significant, multifaceted challenges in delivering geriatric care. This divergence suggests that while clinicians feel competent in isolated diagnostic tasks, they encounter severe difficulties navigating the broader complexities inherent to ageing populations, including patient cognitive decline, physical frailty, multimorbidity, and compromised compliance.

To mitigate these challenges, respondents consistently indicated a preference for comprehensive, blended continuing education (61.8%). Isolated theoretical seminars were deemed insufficient; instead, practitioners expressed a distinct need for multifaceted programs that integrate hands-on workshops, online resources, and structured peer collaboration. This preference is supported by emerging evidence demonstrating the effectiveness of digital communication and educational technologies in improving oral health knowledge and engagement [47]. Furthermore, chatbot-assisted educational interventions have shown promising results as complementary tools for clinical training and patient education [48].

The growing emphasis on practical and technology-enhanced learning is also consistent with established technology acceptance frameworks. Studies utilizing the Unified Theory of Acceptance and Use of Technology (UTAUT) have demonstrated that perceived usefulness, ease of use, and behavioral intention are significant determinants of teledentistry adoption [49]. Similar findings have been reported among underserved populations, where acceptance of remote oral healthcare services is influenced by accessibility and perceived value [50]. Patient-centered investigations have likewise shown that individuals generally appreciate the convenience and accessibility offered by teledentistry while continuing to value direct clinician interaction when appropriate [51]. Collectively, these findings reflect an industry-wide demand for practical, workflow-integrated training capable of bridging the gap between theoretical knowledge and real-world clinical competence.

### 4.5. The Financial Baseline: Systemic Constraints in Geriatric Care Optimisation

Ultimately, the successful deployment of technological innovations and collaborative clinical models is strictly bound by macro-level socioeconomic and systemic constraints. Although participants’ strategic priorities for improving geriatric care were highly fragmented among public awareness, accessibility, and specialised programs, inferential modelling revealed no statistically significant correlation between these specific strategic paths and the perceived importance of financial support (r = 0.065, *p* = 0.551). Rather than indicating an absence of financial concern, this lack of statistical significance underscores that financial support is viewed as a uniform, baseline necessity that transcends individual strategic preferences.

This systemic consensus is heavily reinforced by descriptive data, where 52.4% of respondents cited financial limitations as the primary barrier to optimal care delivery. Previous economic evaluations have demonstrated that teledentistry can reduce costs while simultaneously improving service accessibility [52]. Similar findings have been reported in earlier assessments of telehealth-based dental programs, which documented significant economic benefits associated with remote consultation models [53]. More recent reviews focusing on rural populations have further highlighted the capacity of teledentistry to improve access while reducing healthcare disparities and associated costs [54].

At the same time, sustainable implementation requires more than technological availability alone. Research exploring professional perspectives on sustainable dentistry indicates that long-term success depends on organisational commitment, resource allocation, and supportive policy environments [55]. Technological advances continue to expand clinical possibilities, including the use of quantitative light-induced fluorescence for enhanced detection and monitoring of oral conditions [56]. Likewise, digital workflows have transformed fixed implant prosthodontics [57], while contemporary digital impression systems have significantly improved efficiency and accuracy within restorative and prosthodontic practice [58].

Despite these advances, barriers to adoption remain particularly relevant among older adults. Reviews examining facilitators and obstacles to teledentistry implementation in geriatric populations have identified technological literacy, accessibility, infrastructure, and user confidence as recurring challenges [59]. Patient-focused investigations have similarly demonstrated that acceptance of teledentistry varies according to individual demographic, clinical, and technological factors [60]. Financing remains an especially important issue within long-term care systems, where oral healthcare services are frequently underfunded and inconsistently integrated into broader healthcare delivery models [61].

The persistence of these barriers is further reflected in studies examining dentists’ experiences with teledentistry during and following the COVID-19 pandemic. While many practitioners reported positive attitudes toward digital technologies, concerns regarding implementation, reimbursement, training, and infrastructure remained prevalent [62]. Comparable findings have been reported in investigations assessing dentists’ knowledge and attitudes toward teledentistry [63]. End-user acceptance studies have demonstrated that usability, perceived usefulness, and organisational support are critical determinants of successful adoption [64], while evaluations of web-based diagnostic systems emphasise the importance of reliability and user-centered design for sustained clinical use [65].

This framework directly mirrors patient-level constraints where treatment decisions are heavily dictated by cost, comprising 14.3% for cost alone and 42.8% for cost combined with functionality or aesthetics. These findings imply that professional willingness, clinical confidence, and technological availability are necessary but insufficient components of healthcare optimisation. Without targeted systemic funding to subsidise digital infrastructure and reduce patient-side financial burdens, the gap between perceived clinical importance and the actual delivery of care will remain unbridged. This conclusion is further reinforced by recent scoping reviews indicating that digital health technologies possess considerable potential to improve oral healthcare delivery and support healthy ageing, provided that educational, financial, and infrastructural barriers are adequately addressed [66].

### 4.6. Scope, Generalizability, and Methodological Caveats

This study is subject to several limitations that warrant consideration when interpreting the findings. First, the relatively small sample size (N = 84) necessitated the collapsing of certain data categories to preserve statistical validity, thereby preventing highly granular, subgroup-level inferential analyses. Second, the heavy predominance of European respondents (90.5%) limits the broader geographic generalizability of the results to regions with differing healthcare infrastructures, reimbursement models, or digital dental adoption rates. Third, the reliance on self-reported survey data introduces the inherent risk of response bias, as participants may overreport or underreport clinical behaviours based on perceived professional norms. Finally, recruitment through professional and alumni networks may have introduced a degree of selection bias, potentially favouring participants who already possess a heightened interest or familiarity with digital technologies.

Accordingly, these findings should be interpreted as reflecting subjective professional perceptions rather than objective clinical outcomes. Notwithstanding these limitations, the study provides preliminary insights into patterns of professional technology adoption that may inform future research and implementation strategies. Ultimately, while personal health experiences did not appear to significantly shape beliefs, active engagement in interdisciplinary synergy directly correlated with a higher valuation of its importance. This suggests that collaborative practices should be fostered early and consistently, provided that financial resources are available to bridge the gap between perceived importance and actual clinical practice.

## 5. Conclusions

This study highlights the transformative potential of teledentistry in addressing the growing demands of geriatric oral healthcare, while also underscoring the gap between technological capability and real-world implementation. Although healthcare professionals recognise the value of digital tools and interdisciplinary collaboration, adoption remains inconsistent due to structural, educational, and economic barriers [19,20,62]. The findings emphasise that technological innovation alone is insufficient; meaningful improvements require integration within a comprehensive, patient-centered care model [22,26]. Technology adoption is influenced by career stage dynamics and is most effectively sustained when users surpass the initial one-year adjustment period, after which integration becomes stable and routine. While personal experiences have minimal impact on beliefs, fostering collaboration and ensuring adequate financial support are essential to translating perceived value into consistent professional practice.

The present study explored perceptions and self-reported practices rather than objective clinical outcomes; therefore, the findings should be interpreted with appropriate caution. Future prospective studies may benefit from incorporating additional measures, such as prosthetic success rates and patient-reported outcomes, to provide a more comprehensive understanding of clinical effectiveness. Future research should prioritise longitudinal, multicenter studies to assess the clinical and economic effectiveness of teledentistry and intraoral scanning in geriatric populations [18,37]. Standardised protocols, structured training, supportive policies, and integrated digital platforms enabling real-time interdisciplinary collaboration are essential [11,31,54,64]. Emphasis on patient-centered outcomes, alongside advances in AI, mobile diagnostics, and remote monitoring, can establish teledentistry as a sustainable, accessible, and high-quality approach for geriatric oral healthcare [6,7,27,36,65,66].

## Figures and Tables

**Table 1:** Data Collection and Study Variables.

Study Variables	Variables
Data Collection Method	Anonymous online questionnaire
Independent Variables	Age group; sex; educational level; specialty; years of experience; type of practice; geographical region; proportion of older patients; frequency of clinical engagement with older adults
Outcome Variables	Clinical practice patterns; use of intraoral scanning technologies; assessment of inflammatory oral conditions; interdisciplinary collaboration; perceived effectiveness of teledentistry; perspectives on research and innovation
Measurement Scales	Likert-scale items (attitudes, perceptions); categorical responses; open-ended questions
Qualitative Component	Thematic exploration of open-ended responses

**Table 2:** Demographic Characteristics of the Study Sample (*N* = 84).

Demographic Characteristics	Variable	*N*	%
Geographical Location	Europe	76.0	90.5
	Other regions	8.0	9.5
Age group (years)	50–59	28	33.3
	20–29	25	29.8
	30–39	17	20.2
	40–49	11	13.1
	60–69	3	3.6
Gender	Male	44	52.4
	Female	40	47.6
Educational Level	DDS/DMD	39	46.4
	Postgraduate (MS/MClinDent)	30	35.7
	PhD	13	15.5
	Other	2	2.4
Specialty	General Dentistry	31	36.9
	Prosthodontics	22	26.2
	Νurse	11	13.1
	Endodontics	5	6.0
	Oral Surgery	5	6.0
	Geriatric dentistry	3	3.6
	Other specialties	7	8.2
Years of Experience	>21 years	32	38.1
	0–5 years	28	33.3
	6–10 years	10	11.9
	16–20 years	8	9.5
	11–15 years	6	7.1
Work Setting	Private Practice	36	42.9
	Private + Academic	21	25.0
	Hospital	12	14.3
	Academic	9	10.7
	Hospital + Private	6	7.2
Patients ≥ 65 years in Practice	≤50% of patient base	60	71.4
	>50% of patient base	24	28.6

**Table 3:** Clinical Practice Characteristics Related to Geriatric Patients (*N* = 84).

Clinical Practice Characteristics Related to Geriatric Patients	Variable	*N*	%
Frequency of Monitoring Geriatric Patients	Daily	49	58.3
	Weekly	27	32.1
	Other frequencies	8	9.6
Primary Oral Health Concerns	Tooth loss + Periodontal disease	36	42.9
	Tooth loss	18	21.4
	Tooth loss + Periodontal disease + Xerostomia	16	19.0
	Periodontal disease	5	6.0
	Other	9	10.7
Perceived Impact of Chronic Inflammation	Significantly adverse	36	42.9
	Moderately adverse	39	46.4
	Total negative impact	75	89.3
Methods for Assessing Inflammatory Conditions	Clinical exam + Radiographic imaging	44	52.4
	Clinical exam only	13	15.5
	Clinical exam + Radiographic + Intraoral scanning	12	14.3
	Other methods	15	17.8
Impact on Prosthetic Treatment	Believe chronic inflammation negatively affects outcomes	79	94.0
	Do not/uncertain	5	6.0

**Table 4:** Utilisation of Intraoral Scanning and CAD/CAM Technology (*N* = 84).

Utilisation of Intraoral Scanning and CAD/CAM Technology	Variable	*N*	%
Duration of Intraoral Scanner Use	<1 year	52	61.9
	1–3 years	23	27.4
	>3 years/other	9	10.7
Perceived Effect on Inflammation Management	No effect	38	45.2
	Moderate improvement	20	23.8
	Significant improvement	26	31.0
	Overall improvement (moderate + significant)	46	54.8
CAD/CAM Prosthetic Applications	Fixed Partial Dentures + Implant-supported prostheses	28	33.0
	All restorations (complete, partial, fixed, implant-supported)	11	13.1
	Implant-supported prostheses only	10	11.9
Perceived Advantages of CAD/CAM	No CAD/CAM use for listed restorations	15	17.9
	Other combinations	20	24.1
	Reduced chair time	23	27.4
	All benefits equally important	13	15.5
	No perceived advantage	10	11.9
	Other advantages (fit, aesthetics, precision)	38	5.2
Barriers to Implementation	Cost	20	23.8
	Technical limitations	15	17.9
	Cost + Technical limitations	11	13.1
	Other barriers	38	45.2

**Table 5:** Interdisciplinary Approach (*N* = 84).

Interdisciplinary Approach	Variable	*N*	%
Current Collaboration with Specialists	No	51	60.7
	Yes	33	39.3
Perceived Importance of Interdisciplinary Care	Very important	44	52.4
	Important	33	39.3
	Neutral	7	8.3
Role of Prosthodontics in Geriatric Care	Important	48	57.1
	Critical	28	33.3
	Moderate	7	8.3
	Minimum	1	1.2

**Table 6:** Future Research and Innovations (*N* = 84).

Variable	Category	*N*	%
Impact of Intraoral Scanning on Gerodontology	High impact	37	44.0
	Moderate impact	33	39.3
	Low impact	13	15.5
	No impact	1	1.2
Interest in Research Participation	Yes	53	63.1
	No	31	36.9
Priority Research Areas Variable Impact of Intraoral Scanning on Gerodontology	Interdisciplinary approaches	51	60.8
	Long-term treatment outcomes	28	33.3
	Digital technologies (Intraoral scanning/CAD-CAM)	21	25.0
	Patient collaboration	24	28.6
	Other	26	30.9
	Category	N	%
	High impact	37	44.0
	Moderate impact	33	39.3

**Table 7:** Professional Perspectives and Confidence (*N* = 84).

Professional Perspectives and Confidence	Variable	*N*	%
Confidence in Diagnosing Inflammation	Moderately confident	46	54.8
	Very confident	21	25.0
	Low confidence	17	20.2
Preferred Training Methods	Continuing education courses	20	23.8
	Combined approaches (courses, workshops, web, collaboration)	19	22.6
	Education + collaboration	14	16.7
	Other methods	31	36.9
Challenges in Geriatric Care	Have experienced challenges	80	95.2
	No challenges	4	4.8

**Table 8:** Patient Beliefs (*N* = 84).

Patient Beliefs	Variable	*N*	%
Perceived Importance of Oral Health (Patients)	High importance	35	41.7
	Neutral	16	19.0
	Low importance	26	30.9
	Other	7	8.4
Determinants of Treatment Decisions	Cost only	12	14.3
	Cost + Functionality	19	22.6
	Cost + Functionality + Aesthetics	17	20.2
	Other combinations	36	42.9

**Table 9:** Strategic Priorities and System Needs (*N* = 84).

Variable	Category	*N*	%
Strategic Priorities	Awareness, accessibility, and specialized geriatric services	64	76.2
	Comprehensive/multiple strategies and other responses	20	23.8
Factors for Effective Care	Individual factors (financial support, training, collaboration)	48	57.1
	Comprehensive approach and other responses	36	42.9

**Table 10:** Evaluation of Clinical Practice (*N* = 84).

Variable	Main Finding	*N*	%
Satisfaction with geriatric care capacity	Satisfied	49	58.3
Perceived effectiveness of intraoral scanning	Effective	43	51.2
Preferred prosthetic success assessment	Multidimensional evaluation	53	63.1
Preferred continuing education approach	Formal/professional education methods	43	51.2
Main barriers to geriatric care	Financial and access-related barriers	60	71.4
Observed changes in patient needs	Yes	69	82.1
Willingness to participate in future research	Yes	65	77.4

## Data Availability

The data that supports the findings of this study are available from the corresponding author upon reasonable request, subject to applicable ethical and data protection considerations.

## Abbreviations

The following abbreviations are used in this manuscript:

AUTH	Aristotle University of Thessaloniki
CAD	Computer-Aided Design
CAM	Computer-Aided Manufacturing
DDS	Doctor of Dental Surgery
DMD	Doctor of Dental Medicine
MSc	Master of Science
PhD	Doctor of Philosophy
PROs	Prosthodontics

## Appendix A. Written Informed Consent

ARISTOTLE UNIVERSITY OF THESSALONIKI

SCHOOL OF DENTAL MEDICINE

PROSTHODONTICS LABORATORY

Dear Sir/Madam,

We would like to invite you to participate in an international research study conducted by the Prosthodontics Laboratory of the School of Dental Medicine at Aristotle University of Thessaloniki, titled “Utilising Teledentistry for an Interdisciplinary Approach to Elderly Patients.” This study aims to integrate Teledentistry and interdisciplinary collaboration into the care of elderly patients undergoing prosthetic treatment.

The purpose of this study is to investigate the use of intraoral and extraoral scanning technologies and Teledentistry, which enables remote diagnosis and monitoring, ensuring better access to care for elderly patients, who often face mobility challenges. Furthermore, the study emphasises the cooperation between various healthcare professionals, such as dentists, geriatricians, and physicians from other disciplines, in order to provide comprehensive care to elderly patients.

Your participation involves completing an online questionnaire with 40 questions, which will take approximately 20–25 min to complete. The study’s results will be used for research purposes and may be presented anonymously in scientific journals or conferences.

Participation is voluntary, and you may withdraw at any time without consequences. We assure you that all responses will remain confidential.

If you have any questions, please don’t hesitate to contact Dr Panagiota Chatzidou via email at [email] or by phone at [phone number].

Sincerely,

Panagiota Chatzidou—Principal Investigator

Vasiliki Anastasiadou, Member of the Investigation Team

Olga Naka—Scientific Supervisor

## Appendix B

**Table A1:** Investigation Questionnaire “Utilising Teledentistry for an Interdisciplinary Approach to Elderly Patients”.

A. Demographics	
1. Please indicate your age	20–29
	30–39
	40–49
	50–59
	60–69
	70 years or older
2. Please specify your gender:	Male
	Female
	Other
3. Please indicate your highest level of education attained	DDS/DMD
	Postgraduate Degree (MSc, MC Lin Dent)
	Postgraduate Degree (PhD)
	Other (please specify)
4. Please specify your specialty (or subspecialty)	
5. What is your total number of years of experience?	0 to 5 years of experience
	6 to 10 years of experience
	11 to 15 years of experience
	16 to 20 years of experience
	More than 20 years of experience
6. What type of practice are you presently engaged in?	Private practice
	Academic institution
	Hospital/clinic
	Research facility
	Other (please specify)
7. You practice in	Europe
	UK
	Asia
	USA
	Canada
	Other (please specify)
8. What is the average percentage of your patients who are 65 years of age or older?	0–25%
	26–50%
	51–75%
	76–100%
B. Clinical Practice	
9. How frequently do you engage with geriatric patients in your professional practice?	Daily occurrence
	Weekly occurrence
	Monthly occurrence
	Rare occurrence
	Other (please specify)
10. What are the predominant oral health concerns observed in elderly patients? (Please select all that are applicable)	Tooth loss
	Periodontal disease
	Oral mucosal lesions (please specify)
	Xerostomia
	Oral Candidiasis
	Other (please specify)
11. Based on your expertise, how does chronic inflammation, often referred to as inflammaging, impact the oral health of elderly patients?	Significantly adverse
	Moderately adverse
	No discernible effect
	Moderately positive effect
	Significantly positive effect
12. What methods do you utilise to assess the oral inflammatory conditions of elderly patients? (Please select all applicable options)	Clinical Examination
	Radiographic Imaging
	Intraoral Scanning
	Salivary Markers
	Swabs
	Other (Please Specify)
13. Do you believe that chronic inflammation impacts the effectiveness of prosthodontic treatments?	Yes
	No
	Unsure
C. Utilisation of Intraoral Scanning Technology	
14. Are you currently utilising intraoral scanning technology in your practice?	Yes
	No
15. For what duration have you been utilising Intraoral Scanning Technology?	Less than one year
	One to three years
	Four to six years
	More than six years
16. What is your evaluation of the influence of intraoral scanning technology on the management of inflammation in elderly patients?	Significantly improves
	Moderately improves
	No effect
	Moderately worsens
	Significantly worsens
17. What categories of prosthetics do you typically fabricate utilising computer-aided design and computer-aided manufacturing (CAD/CAM)? (Please select all that are applicable)	Complete dentures
	Partial dentures
	Fixed dental prostheses
	Implant-supported prostheses
	Other (please specify)
	N/A
18. What are the primary advantages of CAD/CAM technology for elderly patients? (Please select all relevant options.)	Improved fit
	Reduced chair time
	Enhanced aesthetics
	Greater precision
	Other (please specify)
	N/A
19. What challenges do you encounter when utilizing CAD/CAM technology in the care of elderly patients? (Please select all applicable options)	Technical limitations
	Patient compliance
	Cost
	Training
	Other (please specify)
D. Interdisciplinary Approach	
20. Do you collaborate with other healthcare professionals, such as geriatricians and immunologists, to provide care for elderly patients?	Yes
	No
21. What is your assessment of the significance of an interdisciplinary approach in managing oral health among older adults?	Very important
	Important
	Neutral
	Unimportant
	Very unimportant
22. What significance do you ascribe to prosthodontics in the comprehensive health management of geriatric patients?	Critical
	Important
	Moderate
	Minimal
	None
E. Future Research and Innovations	
23. Do you foresee that Intraoral Scanning Technology will influence the field of geriatric dentistry?	Major impact
	Moderate impact
	Minor impact
	No impact
24. Would you be interested in participating in research concerning Intraoral Scanning Technology and geriatric oral health?	Yes
	No
25. Which research domains necessitate enhanced attention in geriatric dentistry? (Please select all that are applicable)	Interdisciplinary approach
	Intraoral Scanning Technology
	CAD/CAM technology
	Patient compliance
	Long-term outcomes of prosthodontic treatments
	Other (please specify)
F. Individual Reflections	Highly confident
26. To what extent do you feel confident in diagnosing inflammatory conditions in geriatric patients?	Moderately confident
	Neutral
	Slightly unconfident
	Exhibiting significant lack of self-assurance
27. What resources or training programs could enhance your ability to manage geriatric patients? (Please select all that apply)	Courses in continuing education
	Workshops
	Web resources
	Collaboration with specialists
	Other (please specify)
28. Have you encountered any challenges in providing optimal care to elderly patients?	Yes
	No
G. Patient Perspectives	
29. In your opinion, how do elderly patients perceive the significance of oral health in relation to their overall well-being?	Extremely Significant
	Significant
	Neutral
	Of Minor Importance
	Very Minor Importance
	Extremely Significant
30. Which factors influence an elderly patient’s decision to pursue prosthodontic treatment? (Please select all that are applicable)	Cost
	Aesthetics
	Functionality
	Recommendations from healthcare providers
	Other (please specify)
H. Closing Questions	
31. In what ways can the dental community more effectively cater to the needs of the ageing population? (Please select one option)	Increase awareness and education
	Improve accessibility to dental care
	Enhance interdisciplinary collaboration
	Develop specialised geriatric programs
	Other (please specify)
32. Which factors in geriatric patients should be considered? (Please select all that are applicable)	Financial support
	Additional training
	Research funding
	Collaboration with other professionals
	Other (please specify)
33. Do you have any additional comments or insights regarding the correlation between intraoral scanning technology and geriatric oral health?	Yes (please specify)
	No
I. Evaluation	
34. To what extent are you satisfied with the capability of your current practice to provide care for elderly patients?	Very Unsatisfied
	Unsatisfied
	Neutral
	Satisfied
	Very Satisfied
35. How would you evaluate the effectiveness of Intraoral Scanning Technology in improving treatment outcomes for geriatric patients?	Very Ineffective
	Ineffective
	Neutral
	Effective
	Very Effective
36. Which metrics are utilized to evaluate the success of prosthodontic treatment out-comes in elderly patients? (Please select all that apply)	Patient-reported outcomes
	Clinical assessments
	Longevity of prosthetics
	Other (please specify)
37. What is your preferred approach for remaining informed about developments in geriatric dentistry? (Please select all applicable options)	Academic Journals
	Professional Conferences
	Online Educational Courses
	Professional Networking Platforms
	Other (please specify)
38. Would you be willing to participate in forthcoming studies related to geriatric dentistry and intraoral scanning technology?	Yes
	No
39. Have you observed any changes in the oral health needs of your elderly patients over the years?	Yes
	No
40. What is the most significant challenge currently facing geriatric dentistry? (Please select one option)	Accessibility of healthcare services
	Awareness and educational initiatives
	Financial limitations
	Collaborative efforts across disciplines
	Other (please specify)
